# Training on Reporting and Data System (RADS) for Somatostatin-Receptor Targeted Molecular Imaging Can Reduce the Test Anxiety of Inexperienced Readers

**DOI:** 10.1007/s11307-022-01712-6

**Published:** 2022-03-01

**Authors:** Alexander Weich, Takahiro Higuchi, Ralph A. Bundschuh, Constantin Lapa, Sebastian E. Serfling, Steven P. Rowe, Martin G. Pomper, Ken Herrmann, Andreas K. Buck, Thorsten Derlin, Rudolf A. Werner

**Affiliations:** 1 Department of Internal Medicine II and ENETS Center of Excellence, Gastroenterology, University Hospital Würzburg, Würzburg, Germany; 2 Department of Nuclear Medicine, University Hospital Würzburg, Würzburg, Germany; 3 Dentistry and Pharmaceutical Sciences, Okayama University Graduate School of Medicine, Okayama, Japan; 4 Department of Nuclear Medicine, University Hospital Bonn, Bonn, Germany; 5 Nuclear Medicine, Medical Faculty, University of Augsburg, Augsburg, Germany; 6 The Russell H Morgan Department of Radiology and Radiological Science, Division of Nuclear Medicine and Molecular Imaging, Johns Hopkins School of Medicine, Baltimore, MD USA; 7 Department of Nuclear Medicine, University Hospital Essen, Essen, Germany; 8 Department of Nuclear Medicine, Hannover Medical School, Hannover, Germany

**Keywords:** PET/CT, Neuroendocrine tumor, PRRT, Peptide receptor radionuclide therapy, Reporting and data system, SSTR-RADS, RADS

## Abstract

**Purpose:**

For somatostatin receptor (SSTR)-positron emission tomography/computed tomography (PET/CT), a standardized framework termed SSTR-reporting and data system (RADS) has been proposed. We aimed to elucidate the impact of a RADS-focused training on reader’s anxiety to report on SSTR-PET/CT, the motivational beliefs in learning such a system, whether it increases reader’s confidence, and its implementation in clinical routine.

**Procedures:**

A 3-day training course focusing on SSTR-RADS was conducted. Self-report questionnaires were handed out prior to the course (Pre) and thereafter (Post). The impact of the training on the following categories was evaluated: (1) test anxiety to report on SSTR-PET/CT, (2) motivational beliefs, (3) increase in reader’s confidence, and (4) clinical implementation. To assess the effect size of the course, Cohen’s *d* was calculated (small, *d* = 0.20; large effect, *d* = 0.80).

**Results:**

Of 22 participants, Pre and Post were returned by 21/22 (95.5%). In total, 14/21 (66.7%) were considered inexperienced (IR, < 1 year experience in reading SSTR-PET/CTs) and 7/21 (33.3%) as experienced readers (ER, > 1 year). Applying SSTR-RADS, a large decrease in anxiety to report on SSTR-PET/CT was noted for IR (*d* =  − 0.74, *P* = 0.02), but not for ER (*d* = 0.11, *P* = 0.78). For the other three categories motivational beliefs, reader’s confidence, and clinical implementation, agreement rates were already high prior to the training and persisted throughout the course (*P* ≥ 0.21).

**Conclusions:**

A framework-focused reader training can reduce anxiety to report on SSTR-PET/CTs, in particular for inexperienced readers. This may allow for a more widespread adoption of this system, e.g., in multicenter trials for better intra- and interindividual comparison of scan results.

**Supplementary Information:**

The online version contains supplementary material available at 10.1007/s11307-022-01712-6.

## Introduction

The demand for somatostatin receptor (SSTR) imaging with subsequent therapy for neuroendocrine neoplasms (NEN) has rapidly expanded in recent years [1–3], mainly due to encouraging results of the first randomized, controlled trial demonstrating the efficacy and safety of SSTR-targeted peptide receptor radionuclide therapy (PRRT) [4–6]. To conduct such treatment, uptake in putative sites of disease should be confirmed by a preceding SSTR-directed positron emission tomography (PET)/computed tomography (CT) scan [7, 8], and a precise interpretation of such scans is of utmost importance to select appropriate treatment candidates or to risk-stratify patients [9, 10].

NEN are a very rare and heterogenous tumor entity and therefore — outside of specialized centers — nuclear medicine professionals do not have much routine in the interpretation of SSTR-PET/CT in NEN [11, 12]. Lack of experience with this orphan disease is most likely associated with test anxiety and perceived stress for the interpreting nuclear medicine professional. For instance, in conventional radiology, increased stress caused by diagnostic errors can trigger a vicious circle leading to even more false findings [13, 14]. Nonetheless, a correct scan interpretation is vital for the referring oncologist to initiate the most appropriate therapeutic strategy in patients affected with NEN. As such, in order to minimize stress and anxiety in inexperienced readers (IRs), molecular imaging has to advance their training models and provide guiding tools that determine precisely what makes abnormalities different from normal tissue [15].

Partly as a result, the American College of Radiology has established numerous reporting and data systems (RADS) to enable standardized reporting on imaging findings in a wide variety of diagnostic settings [16]. In analogy to the similarities of different RADS systems for specific organs such as TI-RADS for thyroid or LI-RADS for liver [17, 18], a standardized framework for SSTR-PET/CT termed SSTR-RADS was introduced [19]. SSTR-RADS is based on a 5-point scale (from 1 = no evidence of disease and definitively benign to 5 = high certainty that NEN is present), and is predicated upon the site and intensity of radiotracer uptake [19]. A recent study demonstrated that SSTR-RADS may be useful to identify patients eligible for PRRT [20]. Moreover, a high interobserver agreement rate for SSTR-RADS on scan interpretation was also observed, even among readers with less experience in reading SSTR-PET/CTs [21] and therefore, one may speculate that such a standardized framework system is readily applicable for larger clinical trials or clinical routine in a busy PET/CT practice.

However, little is known on the impact of such a standardized framework on reader’s anxiety to report on SSTR-PET/CTs, the motivational beliefs in learning such a system, whether it increases reader’s confidence, or its implementation in clinical routine. Organized by the European School of Multimodality Imaging and Therapy (ESMIT), a 3-day training course focusing on SSTR-RADS for nuclear medicine professionals was conducted, which allowed the participants to apply SSTR-RADS in a practical manner. Using dedicated questionnaires, we aimed to elucidate the impact of this RADS-focused educational intervention on test anxiety, the motivation to learn SSTR-RADS, the change in level of confidence when applying SSTR-RADS, and the rate of clinical implementation by participants at their home institutions.

## Materials and Methods

### Training

This project was approved by the ESMIT of the European Association of Nuclear Medicine (EANM). All training participants gave written informed consent to participate in this study (including follow-up via email). In May 2018, a 3-day training focusing on SSTR-directed imaging and therapy entitled “Theranostics – clinical and non-clinical aspects (radiopharmacy, physics, and dosimetry) of NENs” was conducted and hosted under the umbrella of the “ESMIT Spring School.” This track was rated as Level 2 within the ESMIT [22]. In brief, this course aimed to cover clinical aspects of molecular imaging and PRRT of NEN patients, while non-clinical topics related to patient management were also addressed. Clinical sessions focused on SSTR-RADS 1.0 [19], including an overview of the most common pitfalls and artifacts in interpreting SSTR-PET/CTs [19, 23, 24] (Table S1). In addition, multiple hands-on cases with different levels of difficulty including respective information on patient’s history were also presented. Provided scans were displayed on a dedicated workstation, thereby allowing to modify the uptake levels. First, participants familiarized themselves with a case displaying normal biodistribution. The second case included a pancreatic NET with a Ki67 of 5–10% and SSTR-RADS scores on a target-lesion level had to be provided by the participants (e.g., lesions classified as SSTR-RADS 1B, SSTR-RADS 4, or SSTR-RADS 5). This respective case was also eligible for SSTR-directed PRRT. Last, a patient afflicted with an atypical carcinoid was presented. On SSTR-PET/CT, the participants had to identify an SSTR-RADS 3D lesion in the lung. A respective [18F]-fluorodeoxyglucose PET/CT was then also displayed, revealing an intense uptake in the SSTR-negative (RADS 3D) lesion (“flip flop phenomenon”). This patient was then considered not eligible for PRRT.

#### Questionnaires and Measures

In total, three questionnaires were provided to the participants. The first questionnaire was handed out prior to the course (Pre), while the second questionnaire was handed out directly after the lectures (Post). Finally, a follow-up (FU) questionnaire was sent out 3 months later via email. Fig. S1 provides an overview of the study design. The participants indicated general information including primary place of work (academic institution or medical center), experience with SSTR-targeted imaging (< 1 year or > 1 year), and whether they perform PRRT at their home institution. All participants are referred to as all readers (AR) in the remainder of the manuscript. Participants were also categorized as IR having < 1 year experience in interpreting SSTR-PET/CTs and experienced readers (ER, > 1 year). All attendees responded to one of the following 12 questions (Q), which were modified from [25] and divided into four categories:

- *Q1–3*, which included items on test anxiety to provide a written report on SSTR-PET/CT;
- *Q*4*–*5, which referred to the motivation to learn a standardized reporting system for SSTR-PET/CT;
- *Q6–8,* which measured the level of reader’s confidence for reading and interpreting SSTR-PET/CT;
- *Q9–12* evaluating the rate of implementation of a standardized framework for SSTR-targeted molecular imaging in clinical routine (Table 1).

**Table 1:** Overview of all questions used in the three questionnaires. PRE was handed out prior to, POST directly thereafter, and FU 3 months after attending the course (distributed via e-mail). *modified according to [20]

	Question No	PRE Questionnaire	POST Questionnaire	FU Questionnaire
**Test** **anxiety**	1*	I have an uneasy, upset feeling when I have to write a written report for a SSTR-PET/CT	I have an uneasy, upset feeling when I have to write a written report for a SSTR-PET/CT	I have an uneasy, upset feeling when I have to write a written report for a SSTR-PET/CT
	2*	I worry a great deal when other clinicians or colleagues ask me about my written SSTR-PET/CT report	I worry a great deal when other clinicians or colleagues ask me about my written SSTR-PET/CT report	I worry a great deal when other clinicians or colleagues ask me about my written SSTR-PET/CT report
	3*	When I write a written report about SSTR-PET/CT I think about how poorly I am doing	When I write a written report about SSTR-PET/CT I think about how poorly I am doing	When I write a written report about SSTR-PET/CT I think about how poorly I am doing
**Motivational beliefs**	4*	I am motivated to learn a standardized reporting system for SSTR-PET/CT	I am motivated to learn a standardized reporting system for SSTR-PET/CT	I am motivated to learn a standardized reporting system for SSTR-PET/CT
	5*	I have a high interest in learning a standardized reporting system for SSTR-PET/CT	I have a high interest in learning a standardized reporting system for SSTR-PET/CT	I have a high interest in learning a standardized reporting system for SSTR-PET/CT
**Level of** **confidence**	6	I think that learning a standardized reporting system for SSTR-PET/CT will have a great impact on my performance reading SSTR-PET/CT	I think that the learned standardized reporting system for SSTR-PET/CT will have a great impact on my performance reading SSTR-PET/CT	I think that the learned standardized reporting system for SSTR-PET/CT had a great impact on my performance reading SSTR-PET/CT scans
	7	I think that learning a standardized reporting system for SSTR-PET/CT will increase my level of confidence when interpreting SSTR-PET/CT scans	I think that the learned standardized reporting system for SSTR-PET/CT will increase my level of confidence when interpreting SSTR-PET/CT scans	I think that the learned standardized reporting system for SSTR-PET/CT increased my level of confidence reading a SSTR-PET/CT
	8	Learning a standardized reporting system for SSTR-PET/CT will help me in differentiating pathological from physiologcial lesion	The learned standardized reporting system for SSTR-PET/CT will help me in differentiating pathological from physiologcial lesion	The learned standardized reporting system for SSTR-PET/CT helped me over the last months in differentiating pathological from physiologcial lesion
**Implementation in** **clinical routine**	9	I think that a standardized reporting system for SSTR-PET/CT will help me considering a treatment with either “cold “ somatostatin analogs or Peptide Receptor Radionuclide Therapy	I think that the learned standardized reporting system for SSTR-PET/CT will help me considering a treatment with either “cold “ somatostatin analogs or Peptide Receptor Radionuclide Therapy	I think that the learned standardized reporting system for SSTR-PET/CT helped me considering a treatment with either “cold “ somatostatin analogs or Peptide Receptor Radionuclide Therapy
	10	I think that learning a standardized reporting system for SSTR-PET/CT will support me in recommending Peptide Receptor Radionuclide Therapy, e.g. in Tumor Boards	I think that the learned standardized reporting system for SSTR-PET/CT will support me in recommending Peptide Receptor Radionuclide Therapy, e.g. in Tumor Boards	I think that the learned standardized reporting system for SSTR-PET/CT supported me in recommending a Peptide Receptor Radionuclide Therapy, e.g. in a Tumor Board
	11	I think that learning a standardized reporting system for SSTR-PET/CT would facilitate my communication with the referring clinician	I think that the learned standardized reporting system for SSTR-PET/CT will facilitate my communication with the referring clinician	I think that the learned standardized reporting system for SSTR-PET/CT facilitated communication with the referring clinician
	12	I think that a standardized reporting system for SSTR-PET/CT will be part of my written reports	I think that the learned standardized reporting system for SSTR-PET/CT will be part of my written reports	The learned standardized reporting system for SSTR-PET/CT is part of my written reports

Independent of the wording that was used, a 4-point Likert scale for self-reflection was provided for all 12 questions (i.e., the answers ranged from 1, “very untrue of me”; 2, “untrue of me”; 3, “true of me”; 4, “very true of me”). In addition, the Qs of all three questionnaires had identical content; however, the Qs in Post and FU have been slightly re-phrased to fit the current situation and timing prior to and after the course.

### Participants and Return Rate of Questionnaires

Pre and Post were returned by 21/22 (95.5%). Due to the low response rate of FU (3/21 [14.3%]), this last questionnaire was removed from further analysis. The vast majority of respondents worked in an academic environment (17/21 [81%]), followed by a medical center (4/21 [19%]). In total, 14/21 (66.7%) were rated as IR, with the remaining 7/21 (33.3%) categorized as ER. And 14/20 (70%) indicated that they do not perform PRRT at their home institutions (with one participant not responding to this item).

### Statistics

To allow for a category-based investigation, a combined analysis of the respective Qs allocated to one of the four categories was conducted. Therefore, to test the impact of the training on test anxiety, motivational beliefs, level of confidence, and implementation of SSTR-RADS in clinical routine, the mean scores of their corresponding items were calculated. Variables are expressed as mean ± standard deviation. Given four possible answers ranging from 1 to 4, the theoretical mean is as follows:

$$(Maximum [4]+Minimum [1]) / 2 = 2.5$$

And therefore, 2.5 indicates neither approval nor disagreement. Consequently, values > 2.5 indicate approval, where values < 2.5 indicate disagreement. The 2-tailed paired Student’s *t*-test was used to compare pre-post values. Statistical analyses were performed in R (version 3.6.1, R Core Team, 2019). Evaluating the training-based impact, the effect size Cohen’s *d* was calculated (small, *d* = 0.20; medium, *d* = 0.50; large, *d* = 0.80) [26]. A *P* < 0.05 was considered statistically significant.

## Results

### SSTR-RADS-focused Training Can Reduce Test Anxiety of IR, but not ER

**Fig. 1:**
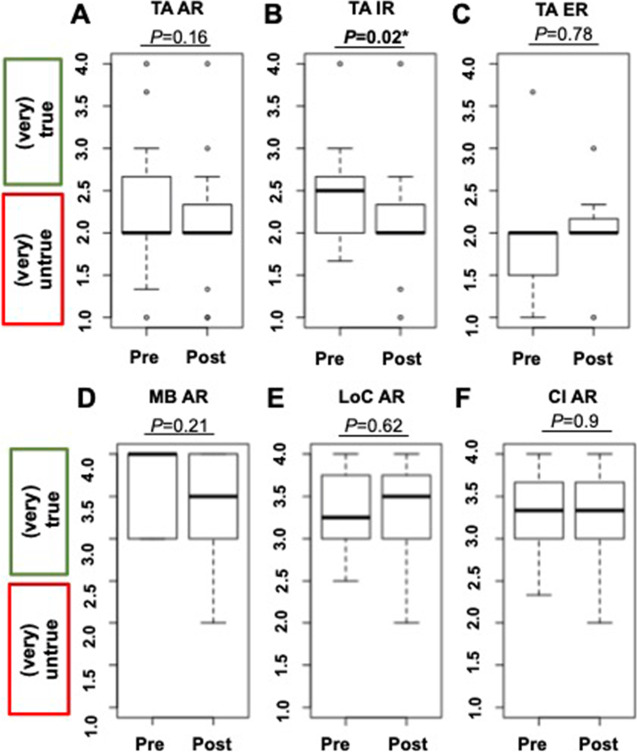
Boxplots showing the comparison of Pre and Post test scores. Values > 2.5 indicate approval, where values < 2.5 indicate disagreement. **A**–**C** displays results for test anxiety (TA). For **A** TA among all readers (AR), a trend towards significant reduction was noted. Significance, however, was reached by inexperienced (IR) (**B**), but not by **C** experienced readers (ER). Among AR, a high approval rate for **D** motivational beliefs (MB), **E** level of confidence (LoC), and **F** rate of clinical implementation (CI) was already recorded prior to the training, which did not change after the intervention. For TA, included items were stated in a negative mode, whereas the remaining categories were phrased in a positive manner, thereby explaining while lower test scores for (**A**–**C**) indicates reduction of TA. In contrast, higher test scores for (**D**–**F**) reflect increase in MB, LoC, and CI. Thick lines indicate median. Data points more than 1.5 times the interquartile range are represented as circles. *reached significance

**Fig. 2:**
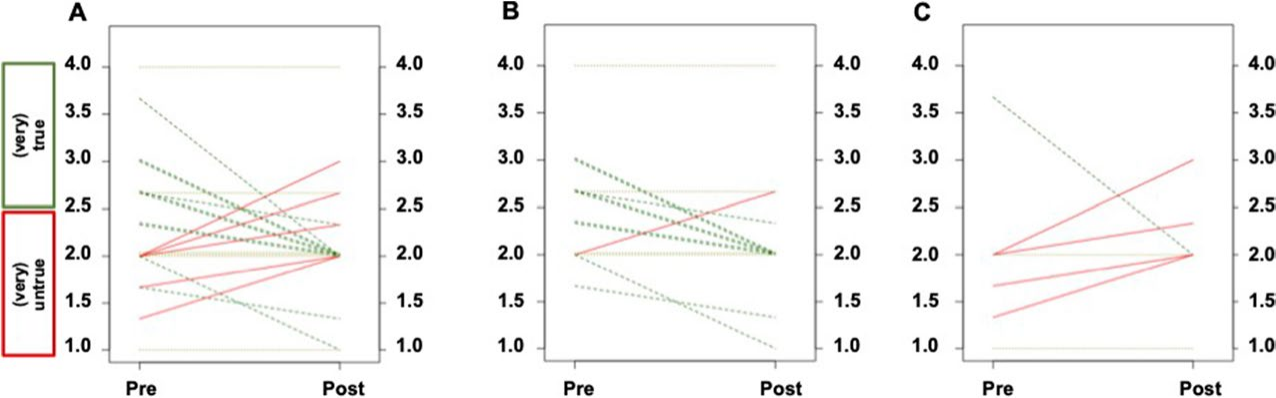
Pre-Post line graphs on test anxiety (referring to questions 1–3 in Table 1) showing the comparison of Pre and Post test scores. For test anxiety, included items were stated in a negative mode, and therefore, green dotted lines indicate reduction and red dotted lines show an increase of test anxiety (dotted ochre lines, no change from Pre to Post). For all readers, 10/21 (47.6%) demonstrated a reduction in test anxiety as indicated by the green lines (**A**), with 9/10 (90%) being categorized as inexperienced participants (**B**), while the remaining 1/10 (10%, **C**) was identified as an experienced individual

Analyzing Q1, Q2, and Q3, with emphasis on the change in test anxiety before and after the training, the change from pre to post on the 4-point Likert scale for AR was − 0.21 ± 0.65, thereby indicating a small to medium effect by the course (*d* =  − 0.32, *P* = 0.16; Fig. 1A). However, this was driven by IR with a significant medium to large reduction in anxiety (change Pre to Post, − 0.36 ± 0.48; *d* =  − 0.74, *P* = 0.02; Fig. 1B), while for ER, only an insignificant effect caused by the program was noted (change, 0.10 ± 0.85, *d* = 0.11; *P* = 0.78; Fig. 1C). This was also further corroborated on an intraindividual level. In total, 10/21 (47.6%) of AR demonstrated a reduction in test anxiety (Fig. 2A, green lines), from whom 9 (90%) were identified as IR (ER, 1/10 [10%]; Fig. 2B, C).

### Throughout the Training, Participants were Motivated to learn SSTR-RADS

**Fig. 3:**
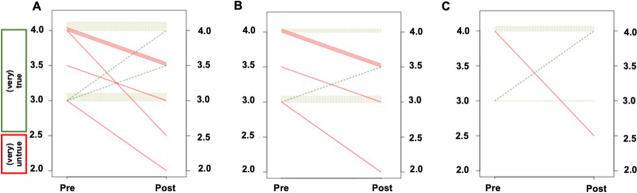
Pre-Post line graphs on motivational beliefs (referring to questions 4 and 5 in Table 1) showing the comparison of Pre and Post test scores. For motivational beliefs, the items were phrased in a positive mode and therefore, green dotted lines indicate an increased motivation to learn SSTR-RADS and red dotted lines show a decreased motivation (dotted ochre lines, no change from Pre to Post). For all readers, 2/21 (9.5%; **A**) reported on an increase in motivation (green lines), with one participant (1/2, [50%]) categorized as inexperienced (**B**) and the other participant (1/2, [50%]) as experienced (**C**). However, already prior to the course, 21/21 (100%) were motivated to learn SSTR-RADS (test score > 2.5)

Analyzing Q4 and Q5, with an emphasis on change in motivation to learn a standardized framework, AR were motivated to learn RADS already prior to the course (Pre, 3.55 ± 0.50), which remained stable (Pre to Post decline, 0.14 ± 0.50; Fig. 1D). Consequently, for AR, only a small course-based effect on motivational beliefs was observed (*d* =  − 0.28, *P* = 0.21). This was also independent of previous reading experience (IR, change Pre to Post, − 0.18 ± 0.37, *d* =  − 0.48, *P* = 0.1; ER, − 0.07 ± 0.73, *d* =  − 0.1, *P* = 0.81). On an intraindividual level, 2/21 (9.5%; Fig. 3A) reported on an increase in motivation (IR and ER, 1/2 [50%], respectively; Fig. 3B, C, green lines). However, already prior to training, 21/21 (100%) were motivated to learn SSTR-RADS (i.e., test score > 2.5; Fig. 3A) and a decline ≤ 2.5 (indicative for less motivation or indifference) was only noted in 2/21 (9.5%) individuals after the program.

### Reader’s Confidence Remains High when Applying SSTR-RADS

**Fig. 4:**
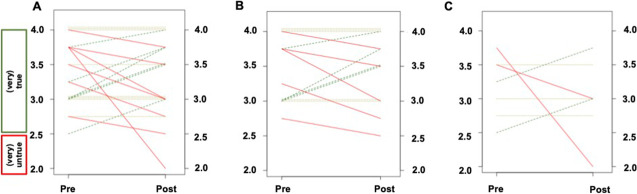
Pre-Post line graphs on level of confidence (referring to questions 6–8 in Table 1) showing the comparison of Pre and Post test scores (derived from questionnaires handed out prior to the training and directly thereafter). For assessing the level of confidence, the items were phrased in a positive mode and therefore, green dotted lines indicate an increase in the level of confidence when SSTR-RADS is applied and red dotted lines show a decline in the level of confidence (dotted ochre lines, no change from Pre to Post). For all attendees, 6/21 (28.6%) demonstrated an increase in confidence (**A**, green lines), which was primarily driven by 4/6 (66.7%) of the inexperienced participants (**B**) when compared to the attendees categorized as experienced (2/6, [33.3%]) (**C**). Again, already prior to the course, 20/21 (95.2%) reported on a high level of confidence (test score > 2.5)

Analyzing Q6, Q7, and Q8 to assess the change in reader’s confidence, AR already indicated prior to the intervention that their level of confidence is high when a standardized framework for SSTR-PET/CT is applied (Pre, 3.36 ± 0.48), which did not change throughout the training (− 0.06 ± 0.54), indicating a small course-based effect (*d* =  − 0.11, *P* = 0.62; Fig. 1E). This was again independent of previous reading experience. IR indicated high approval rates when asked if SSTR-RADS can increase their level of confidence (Pre, 3.45 ± 0.48; change Pre to Post, 00 ± 0.40), with no impact due to the training (*d* = 0, *P* = 1; ER: change Pre to Post, − 0.18 ± 0.77; *d* =  − 0.23, *P* = 0.56). On an intraindividual level, 6/21 (28.6%) demonstrated an increase in confidence (Fig. 4A, green lines; IR, 4/6 [66.7%], ER, 2/6 [33.3%]; Fig. 4B, C). However, already prior to the course, 20/21 (95.2%,) reported on a high level of confidence (test score > 2.5) (Fig. 4A) and a decline ≤ 2.5 (indicative for decline in the level of confidence or indifference) was only recorded in 2/20 (10%).

### Throughout the Training, Rate of Clinical Implementation of SSTR-RADS Remains High

**Fig. 5:**
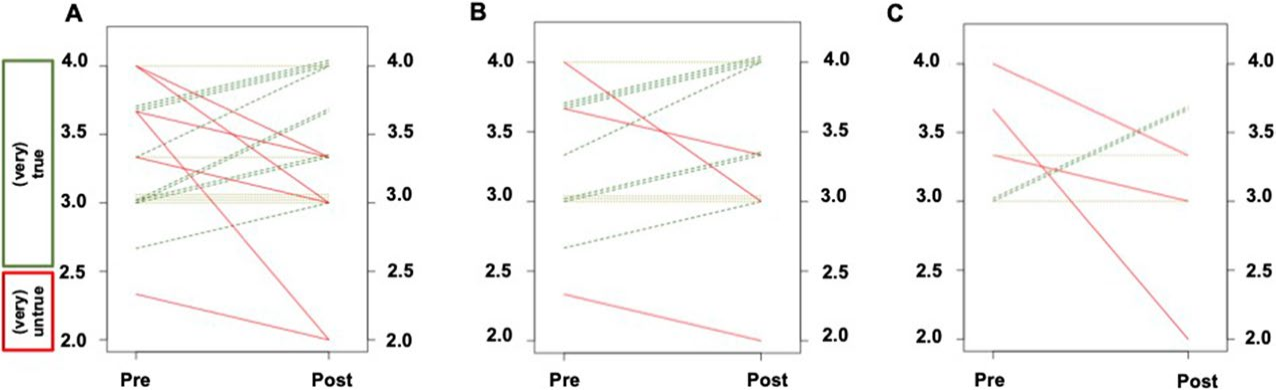
Pre-Post line graphs on rate of clinical implementation (referring to questions 9–12 in Table 1) showing the comparison of Pre and Post test scores (derived from questionnaires handed out prior to the training and directly thereafter). For assessing the rate of clinical implementation, the items were phrased in a positive mode and therefore, green dotted lines indicate an increase in the rate of clinical implementation and red dotted lines show a decrease (dotted ochre lines, no change from Pre to Post). For all readers, 9/21 (42.9%) showed an increase in the rate of clinical implementation of SSTR-RADS at their home institutions (**A**, green line), which was primarily driven by inexperienced readers (**B**) with an approval rate of 7/9 (77.8%) when compared to experienced readers (2/9 [22.2%], **C**). Again, already prior to the course, 20/21 (95.2%) reported on a high level of confidence when SSTR-RAS is applied and a decline < 2.5 (indicative for decrease in the level of confidence) was only recorded in 1/20 (5%, red line, A)

Analyzing Q9, Q10, Q11, and Q12 in order to assess the implementation of SSTR-RADS into clinical routine, AR noted already prior to the course that their rate of implementation is high (Pre, 3.30 ± 0.46), which did not change throughout the program (− 0.02 ± 0.57), corresponding to a small to non-existent effect size due to the training (*d* =  − 0.03, *P* = 0.9; Fig. 1F). When responding to the questionnaire which was handed out prior to the course, IR (Pre, 3.29 ± 0.50) and ER (Pre, 3.33 ± 0.38) recorded that they are willing to use a framework for SSTR-PET/CT at their home institutions, which remained similar throughout the course (IR, 0.07 ± 0.42; ER, − 0.19 ± 0.81). Consequently, the effect size was rated as small for both IR (*d* = 0.17) and ER (*d* =  − 0.23, *P* ≥ 0.53). On an intraindividual level, 9/21 (42.9%) demonstrated an increase in the rate of clinical implementation of SSTR-RADS at their departments (Fig. 5A). This was primarily driven by IR with an approval rate of 7/9 (77.8%; ER, 2/9 [22.2%]; Fig. 5B, C). Already prior to the course, 20/21 (95.2%) reported on a high level of confidence when SSTR-RADS is applied and a decline < 2.5 (indicative for less clinical implementation) was only noted in 1/20 (5%, red line; Fig. 5A).

Table S2 provides an overview of the Likert scale rating before and after the course and the respective changes (along with Cohen’s *d*) for AR. Table 2 provides respective information for IR and Table 3 for ER.

**Table 2:** Overview of the Likert scale rating before and after the training and the respective changes (along with Cohen’s *d*) for inexperienced readers. A significant, medium to large reduction for test anxiety was noted. For motivational beliefs, level of confidence and rate of clinical implementation, pre-/post-interventional test scores remained on a stable high level throughout the training, thereby suggesting a small to medium effect due to the program (as indicated by Cohen’s *d*). *SD*, standard deviation. *Pre*, Questionnaire prior to the course. *Post*, Questionnaire right after the course. * reached significance

Category		Mean ± SD	Median	Range	Change Pre-Post			
					Mean ± SD	Range	Cohen’s *d*	*P*-value
Test anxiety	*Pre*	2.50 ± 0.60	2.50	1.67, 4.00	− 0.36 ± 0.48	− 1.00, 0.67	− *0.74*	0.02*
	*Post*	2.14 ± 0.69	2.00	1.00, 4.00				
Motivational Beliefs	*Pre*	3.46 ± 0.50	3.25	3.00, 4.00	− 0.18 ± 0.37	− 1.00, 0.50	− 0.48	0.1
	*Post*	3.29 ± 0.54	3.25	2.00, 4.00				
Level of Confidence	*Pre*	3.45 ± 0.48	3.50	2.75, 4.00	0.00 ± 0.40	− 0.75, 0.75	0	1.00
	*Post*	3.45 ± 0.51	3.50	2.50, 4.00				
Implementation in the Clinic	*Pre*	3.29 ± 0.50	3.17	2.33, 4.00	0.07 ± 0.42	− 1.00, 0.67	0.17	0.53
	*Post*	3.36 ± 0.59	3.33	2.00, 4.00

**Table 3:** Overview of the Likert scale rating before and after the training and the respective changes (along with Cohen’s *d*) for experienced readers. For test anxiety, no significant reduction was noted, but the experienced respondents declined per se to be anxious when writing a report on a somatostatin receptor-targeted scan. For motivational beliefs, level of confidence, and rate of clinical implementation, pre-/post-interventional test scores remained on a stable high level throughout the training, thereby suggesting a small effect due to the program (as indicated by Cohen’s *d*). *SD*, standard deviation. *Pre*, Questionnaire prior to the course. *Post*, Questionnaire right after the course

Category		Mean ± SD	Median	Range	Change Pre-Post			
					Mean ± SD	Range	Cohen’s d	*P*-value
Test anxiety	*Pre*	1.95 ± 0.85	2.00	1.00, 3.67	0.10 ± 0.85	− 1.67, 1.00	0.11	0.78
	*Post*	2.05 ± 0.59	2.00	1.00, 3.00				
Motivational Beliefs	*Pre*	3.71 ± 0.49	4.00	3.00, 4.00	− 0.07 ± 0.73	− 1.50, 1.00	− 0.10	0.81
	*Post*	3.64 ± 0.63	4.00	2.50, 4.00				
Level of Confidence	*Pre*	3.18 ± 0.45	3.25	2.50, 3.75	− 0.18 ± 0.77	− 1.75, 0.50	− 0.23	0.56
	*Post*	3.00 ± 0.56	3.00	2.00, 3.75				
Implementation in the Clinic	*Pre*	3.33 ± 0.38	3.33	3.00, 4.00	− 0.19 ± 0.81	− 1.67, 0.67	− 0.23	0.56
	*Post*	3.14 ± 0 .57	3.33	2.00, 3.67				

## Discussion

Training on SSTR-RADS for SSTR-directed PET/CT led to a medium to large reduction of test anxiety in less experienced readers for providing scan reports. Motivation of attendees to learn a framework for PET/CT interpretation, their level of confidence, and rate of clinical implementation were already high prior to training (≥ 3.30 ± 0.46), independent of previous reading experience. In light of these increased pre-interventional test scores, analyses of Cohen’s d revealed a small effect (AR, range, *d* =  − 0.32 to − 0.03), thereby suggesting that the impact of the provided training for these categories was low. Nonetheless, given the stable test scores after the intervention with an average decline for AR of only ≥ 0.02, one may speculate that participants are per se motivated to learn a framework for SSTR-directed imaging interpretation, are convinced that such a framework increases their confidence, and, lastly, are willing to implement RADS at their home institutions.

In general, test anxiety is situation-specific and highly depends on intraindividual differences to what extent an anxious individual experiences an examination as a threat [27]. This rather broad definition has been expanded by Zeidner defining multiple levels of test anxiety, including somatic symptoms such as palpitations or sweating, downgrading self-statements about (academic) failure, and behavioral avoidance tendencies such as procrastination [28, 29]. Of note, in an academic environment, increasing test anxiety was tightly linked to debilitating academic output [30] and therefore, one may speculate that an increasing concern about the written report of a scan may also hamper performance of the interpreting radiologist [31]. In the present study investigating the impact of a RADS-specific course on test anxiety in the context of SSTR-PET/CT reporting, an average decline of 0.36 points on a Likert-type scale was noted for IR (*d* =  − 0.74, *P* = 0.02), but not for ER (*d* = 0.11, *P* = 0.78). As reported in the Medscape Radiology Lifestyle Report, recent years have witnessed an alarming trend towards worsening burnout among radiology residents [31, 32]. In this regard, potential risk factors included, but were not limited to concerns about making a medical error [31, 32]. As such, given the encouraging results of anxiety reduction when SSTR-RADS is applied (Fig. 1A–C), one may speculate that this framework may reduce such concerns, thereby potentially contributing to a decrease of professional burnout among radiology trainees.

Motivational beliefs of attendees to learn a framework for SSTR-targeted scan interpretation and their rate of clinical implementation were increased already prior to the course. Of note, for AR, respective Cohen’s *d* was rather low (≥ − 0.28), thereby suggesting only a small impact of the provided training for these categories. Nonetheless, after the course, approval rates for both motivation and clinical implementation remained high (AR, ≥ 3.29 ± 0.58), which further emphasizes that nuclear medicine professionals are eager to learn standardized reporting for SSTR-targeted molecular imaging and are also willing to implement such a system at their departments. In addition, a previous study has already reported on a high interobserver agreement rate, even for less experienced readers, when SSTR-RADS is applied [33]. As such, one may speculate that this or other recently standardized interpretation systems for SSTR-PET/CT will be become more routinely used in multi-center trials [10, 19, 34], which may then allow for better intra- and interinstitutional comparison of scan results [23]. As such, results of the present and previous studies testing SSTR-RADS in different contexts may pave the way for the use of this framework for standardized collection of imaging information for multicenter trials. Nonetheless, prior to a more widespread adoption, further studies are needed, e.g., by correlating framework-based scan findings with histopathological specimen or to test the predictive potential for response to treatment [23]. As such, consensus conferences may arrive at a unified framework, e.g., to apply SSTR-RADS in low-grade NENs and the metabolic grading system in highly proliferative disease [10, 19].

Several limitations have to be considered. First, the number of participants was rather small. Nonetheless, as we chose questionnaires to measure the impact of the training, we considered a medium (0.5) to large effect (0.8) to be of practical relevance [26]. We opted for a pre-post design to achieve higher power to detect training effects [26]. Hence, a power analysis for a two-tailed paired *t*-test with conventional alpha of 0.05, power of 0.8 and a mean of medium to large effect, i.e., Cohen’s *d* of 0.65, resulted in the investigated sample size of *N* = 21. Thus, the number of enrolled participants in our study (*N* = 22) met this requirement. Also, the post hoc grouping of IR and ER led to disproportional subgroups. This may be misleading because the same effect size may be significant in the larger group but not in the smaller group. However, the effect size of test anxiety for ERs was substantially lower than for IRs. Therefore, we consider it rather unlikely that the observed effect for IRs was concealed by the smaller sample size of the ERs. Nonetheless, future studies should aim for larger and more balanced cohorts having different levels of reading expertise or should explicitly incorporate participant’s experience as independent variable to avoid disproportional groups when sampling. Response rate of the follow-up questionnaire was low and thus, follow-up by both postal mail and e-mail may increase return rates [35]. In addition, the high approval rates among the last three categories (reader’s confidence, motivation, and clinical implementation) could also be partially explained by the acquiescence bias, characterized by a tendency towards always being affirmative regardless of the content of the question [36]. To address this, future studies may also implement inherent quality control questions, e.g., whether the attendees are receptive for such a training on interpreting SSTR-PET/CTs.

## Conclusions

A RADS-specific training to interpret SSTR-directed PET/CTs can have a large reduction of test anxiety in less experienced readers. Independent of previous reading experience, motivation of attendees to learn a framework for scan interpretation, their confidence, and their rate of clinical implementation remained stable on a high level throughout the course, thereby suggesting that the impact of the provided training for these categories was low. Nonetheless, given the high pre-/post-interventional approval rates, participants seem to be eager to learn a framework for SSTR-targeted scans, are convinced that such a system increases their level of confidence for interpretation, and are also willing to use RADS at their home institutions. This may allow for a more widespread adoption of this system, e.g., in multicentric trials of NEN patients to collect standardized imaging results for better intra- and interindividual comparison.

## Supplementary Information

Below is the link to the electronic supplementary material.

Supplementary file1 (JPEG 72 KB)

Supplementary file2 (DOCX 33 KB)
