# Supplementary figures and images for: Training on Reporting and Data System (RADS) for Somatostatin-Receptor Targeted Molecular Imaging Can Reduce the Test Anxiety of Inexperienced Readers
Source: Mol Imaging Biol. 2022 Mar 1;24(4):631–40. doi: 10.1007/s11307-022-01712-6 (PMC9296379; doi:10.1007/s11307-022-01712-6)

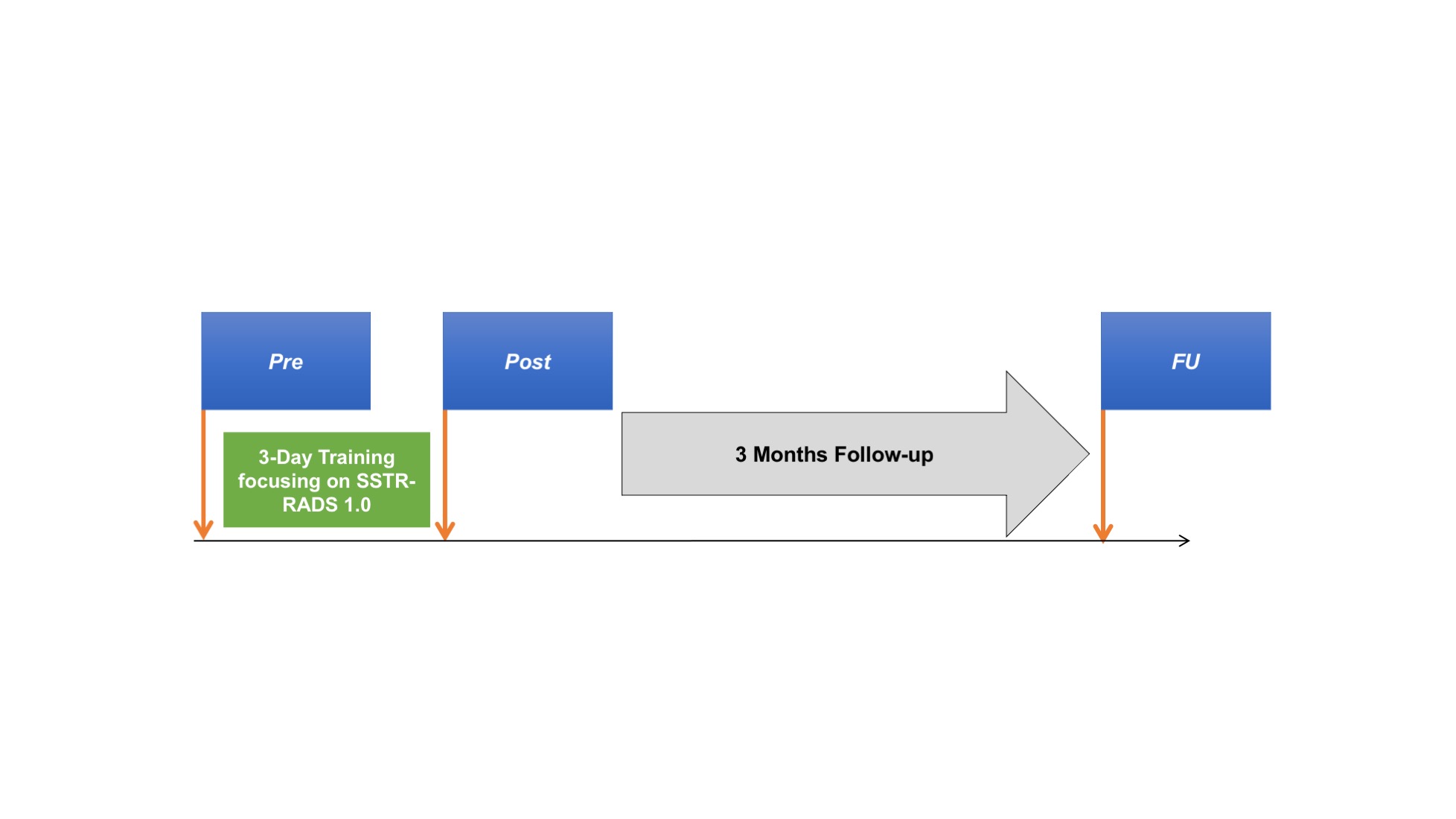

Supplement: Supplementary file 1 — Supplementary file1 (JPEG 72 KB) [file 11307_2022_1712_MOESM1_ESM.jpeg]
