# Supplementary material for: Training on Reporting and Data System (RADS) for Somatostatin-Receptor Targeted Molecular Imaging Can Reduce the Test Anxiety of Inexperienced Readers
Source: Mol Imaging Biol. 2022 Mar 1;24(4):631–40. doi: 10.1007/s11307-022-01712-6 (PMC9296379; doi:10.1007/s11307-022-01712-6)
Supplement: Supplementary file 2 — Supplementary file2 (DOCX 33 KB) [file 11307_2022_1712_MOESM2_ESM.docx]

**Training on Reporting and Data System (RADS) for Somatostatin-Receptor Targeted Molecular Imaging Can Reduce the Test Anxiety of Inexperienced Readers**

**SUPPLEMENTARY TABLES**

|  |  | SSTR-RADS | Workup | Uptake Level# | PRRT? |
| --- | --- | --- | --- | --- | --- |
| 1 | 1A | Benign lesion, characterized by biopsy or anatomic imaging without abnormal uptake | n/a | 1 | N |
|  | 1B | Benign lesion, characterized by biopsy or anatomic imaging with abnormal uptake | n/a | 2-3 | N |
| 2 |  | Soft tissue site or bone lesion atypical for metastatic NET | n/a | 1 | N |
| 3 | 3A | Equivocal uptake in soft tissue lesion typical of NET | B, F/U | 1-2 | N |
|  | 3B | Equivocal uptake in bone lesion not atypical of NET | B, F/U | 1-2 | N |
|  | 3C | Intense uptake in site highly atypical of all but advanced stages of NET (i.e., high likelihood of non-NET malignancy or other benign tumor) | B | 3 | N |
|  | 3D | Lesion suggestive of malignancy on anatomic imaging but lacking uptake. 18F-FDG is recommended to rule out potential dedifferentiation | B, F/U | not available | N |
| 4 |  | Intense uptake in site typical of NET but lacking definitive findings on conventional imaging | n/a | 3 | Y |
| 5 |  | Intense uptake in site typical of NET and with definitive findings on conventional imaging | n/a | 3 | Y |

**Table S1. Reporting and data systems (RADS) for Somatostatin receptor (SSTR)-RADS.** n/a = not applicable, Y(es) = peptide receptor radionuclide therapy (PRRT) is recommended, N(o) = PRRT is not recommended. B = Biopsy, F/U = follow-up imaging after 3-6 months. # = uptake level is defined as either level 1 (focal uptake, but ≤ blood pool), level 2 (> blood pool, but ≤ physiologic liver uptake) or level 3 (> physiologic liver uptake). Modified from Werner et al., J Nucl Med 2021, 62, 514-520 [33], © by the Society of Nuclear Medicine and Molecular Imaging, Inc.

| Category | | Mean±SD | Median | Range | Change Pre-Post | | | |
| --- | --- | --- | --- | --- | --- | --- | --- | --- |
|  |  |  |  |  | Mean±SD | Range | Cohen’s d | P-value |
| Test anxiety | ***Pre*** | 2.32±0.72 | 2.00 | 1.00, 4.00 | -0.21±0.65 | -1.67, 1.00 | -0.32 | 0.16 |
|  | ***Post*** | 2.11±0.64 | 2.00 | 1.00, 4.00 |  |  |  |  |
| Motivational Beliefs | ***Pre*** | 3.55±0.50 | 4.00 | 3.00, 4.00 | -0.14±0.50 | -1.50, 1.00 | -0.28 | 0.21 |
|  | ***Post*** | 3.40±0.58 | 3.50 | 2.00, 4.00 |  |  |  |  |
| Level of Confidence | ***Pre*** | 3.36±0.48 | 3.25 | 2.50, 4.00 | -0.06±0.54 | -1.75, 0.75 | -0.11 | 0.62 |
|  | ***Post*** | 3.30±0.56 | 3.50 | 2.00, 4.00 |  |  |  |  |
| Implementation in the Clinic | ***Pre*** | 3.30±0.46 | 3.33 | 2.33, 4.00 | -0.02±0.57 | -1.67, 0.67 | -0.03 | 0.9 |
|  | ***Post*** | 3.29±0.58 | 3.33 | 2.00, 4.00 |  |  |  |  |

**Table S2. Overview of the Likert scale rating before and after the training and the respective changes (along with Cohen’s d) for all readers.** A trend towards significance for test anxiety reduction was noted. For motivational beliefs, level of confidence and rate of clinical implementation, pre-/post-interventional test scores remained on a stable high level throughout the training, thereby suggesting a small effect due to the program (as indicated by Cohen’s d). SD=standard deviation. Pre=Questionnaire prior to the course. Post=Questionnaire right after the course.
